# Group-based medical mistrust and care expectations among black patients seeking addiction treatment

**DOI:** 10.1016/j.dadr.2022.100026

**Published:** 2022-01-18

**Authors:** O. Trent Hall, Nia M. Bhadra-Heintz, Julie Teater, Jennifer Samiec, Jose Moreno, Kamilah Dixon-Shambley, Kara M. Rood, David A. Fiellin, Ayana Jordan

**Affiliations:** a Department of Psychiatry and Behavioral Health, Ohio State University Wexner Medical Center, Talbot Hall 181 Taylor Ave., Columbus, OH 43203, USA; b College of Medicine, The Ohio State University, Columbus, OH, USA; c Department of Obstetrics and Gynecology, Ohio State University Wexner Medical Center, Columbus, OH, USA; d Program in Addiction Medicine, Department of Internal Medicine, Yale School of Medicine, New Haven, CT, USA; e Yale School of Public Health, New Haven, CT, USA; f Department of Psychiatry, Yale School of Medicine, New Haven, CT, USA

**Keywords:** Racial discrimination, Substance-related disorders, Healthcare disparities, Medical mistrust, Substance use treatment, Social justice, GBMMS, group, based medical mistrust scale, OSUWMC, Ohio State Wexner Medical Center, STEPP, substance use, treatment, education and prevention program, GBMMS LOS, lack of support from healthcare providers subscale, GBMMS SUSP, suspicion subscale, GBMMS DISP, group, based health disparities subscale, HIRHW, history of interpersonal racism by healthcare workers

## Abstract

**Background:**

Black patients seeking addiction care experience poorer treatment access, retention, and outcomes when compared to White counterparts. Black patients may have elevated group-based medical mistrust, which has been associated with poorer health outcomes and increased experiences of racism across multiple healthcare contexts. The relationship between group-based medical mistrust and expectations for addiction treatment among Black individuals remains untested.

**Methods:**

A total of 143 Black participants were recruited from two addiction treatment centers in Columbus, Ohio. Participants completed the Group Based Medical Mistrust Scale (GBMMS) and answered questions related to expectations of addiction treatment. Descriptive analysis and Spearman's rho correlations were performed to assess for relationships between group-based medical mistrust and expectations of care.

**Results:**

Group-based medical mistrust in Black patients was associated with self-reported delay in accessing addiction treatment, anticipation of racism during addiction treatment, non-adherence and discrimination-precipitated relapse. However, non-adherence to treatment was least strongly correlated with group-based medical mistrust demonstrating an opportunity for engagement.

**Conclusion:**

Group-based medical mistrust is associated with Black patients’ care expectations when seeking addiction treatment. Use of the GBMMS within addiction medicine to address themes of mistrust in patients, and potential biases in providers, may improve treatment access and outcomes.

## Highlights

• Group-based medical mistrust (GBMMS) is rooted in awareness of past and present structural and interpersonal discrimination.
• GBMMS has previously been linked to health disparities.
• GBMMS is associated with black patients’ expectations of addiction treatment.
• Treatment delays, anticipation of discrimination, lower adherence, and relapse may be related to GBMMS.
• Group-based medical mistrust and care expectations among black patients seeking addiction treatment.

## 1 Background

Black patients seeking addiction treatment in the United States experience health related disparities including delayed presentation to treatment (Gryczynski et al., 2011), reduced access to treatment (Hadland et al., 2017; Lagisetty et al., 2019; Manhapra et al., 2020; Martin et al., 2020; Nina et al., 2014; Schiff et al., 2020), unequal retention in treatment (Guerrero et al., 2013; Samples et al., 2018) and poorer post-treatment outcomes (Lappan et al., 2020; Montgomery et al., 2020). Despite extensive research demonstrating disparities in addiction treatment, more work is needed to understand the complex structural and interpersonal factors involved. Nascent literature has identified group-based medical mistrust as potentially relevant to Black patients’ expectations of addiction treatment (Hall et al., 2021). However, this study failed to test independent associations between group-based medical mistrust and expectations of care, necessitating additional exploration.

Group-based medical mistrust, defined as “a tendency to distrust medical systems and personnel believed to represent the dominant culture in a given society,” has been linked to health disparities (Benkert et al., 2019). Mistrust involves the belief that an entity will not act in the patient's best interests or may work against them (Williamson and Bigman, 2018). Higher rates of group-based medical mistrust are associated with reduced healthcare utilization, less treatment adherence, and poorer physical and mental health outcomes (Moore et al., 2013; Shelton et al., 2010; Street et al., 2009).

Outside addiction medicine, studies have found medical mistrust to be intertwined with experiences of racism. Endorsement of medical mistrust is associated with endorsement of racial discrimination (Galvan et al., 2017; Williamson et al., 2019). Importantly, recent research by our group found that prior experiences of medical racism are associated with greater group-based medical mistrust and poorer expectations regarding addiction treatment (Hall et al., 2021).

To date, associations between group-based medical mistrust and expectations of addiction treatment among Black patients remain untested. The present study aims to determine if group-based medical mistrust relates to poorer expectations of addiction treatment, particularly with regard to delays in care-seeking due to concern for racial discrimination, projected non-adherence, and fears of discrimination-precipitated relapse.

## 2 Methods

### 2.1 Design and study population

Participants were recruited from the Ohio State Wexner Medical Center (OSUWMC) addiction facility Talbot Hall and the OSUWMC Substance Use, Treatment, Education and Prevention Program (STEPP) clinic at McCampbell Hall. Addiction care at Talbot Hall includes comprehensive outpatient (partial hospitalization, intensive outpatient, group and individual counseling and medication management) treatment as well as medically supervised withdrawal for alcohol and substance use disorders. STEPP clinic offers outpatient addiction treatment, obstetric and postnatal care to pregnant people with substance use disorders. Although embedded in a university environment, neither facility is associated with student health and both primarily treat community members in OSUWMC's catchment area. Over 10-months in 2020, trained staff identified potential participants during routine medical assessments. Those describing their race as Black, African American, or Multiracial with Black or African American as a part of their identity were offered participation. Participants were at least 18 years old. Exclusion criteria were inability to consent, read or comprehend the questionnaire, or operate a tablet. Participants completed the survey on a tablet in private examination rooms. They provided verbal consent and were monetarily compensated.

The survey included the GBMMS (Table 1), original questions about expectations of care regarding racial discrimination and addiction treatment, and demographic information. GBMMS includes twelve items which assess race-based medical mistrust (Shelton et al., 2010). The items are divided among subscales (1) Lack of Support from Healthcare Providers; (2) Suspicion of Healthcare Providers and Medicine; and (3) Group-based Disparities in Healthcare. Response options range from 1 to 5 (strongly disagree to strongly agree). A detailed description of procedures and measures is available elsewhere (Hall et al., 2021).

**Table 1:** Group-based medical mistrust scale (GBMMS).

Lack of support from healthcare providers subscale
Doctors and healthcare workers sometimes hide information from patients who belong to my ethnic group
* Doctors have the best interests of people of my ethnic group in mind
I have personally been treated poorly or unfairly by doctors or healthcare workers because of my ethnicity
Suspicion Subscale
People of my ethnic group should not confide in doctors and healthcare workers because it will be used against them
People of my ethnic group should be suspicious of information from doctors and healthcare workers
People of my ethnic group cannot trust doctors and healthcare workers
People of my ethnic group should be suspicious of modern medicine
Doctors and healthcare workers treat people of my ethnic group like “guinea pigs”
Doctors and healthcare workers do not take the medical complaints of people of my ethnic group seriously
Group-based Health Disparities Subscale
*People of my ethnic group receive the same medical care from doctors and healthcare workers as people from other groups
*People of my ethnic group are treated the same as people of other groups by doctors and healthcare workers
*In most hospitals, people of different ethnic groups receive the same kind of care

Note: Items with * were reverse scored. ‘Ethnicity’ used as a proxy for race to maintain consistency with prior research using GBMMS.

Spearman's rho correlations probed associations between GBMMS and expectations of care. All tests were two-tailed and deemed significant at *α* < 0.01. Analyses were performed using SPSS (Version 27.0, SPSS. Inc.). OSUWMC Institutional Review Board approved this study.

## 3 Results

### 3.1 Descriptive analyses

Of 150 individuals offered participation, 145 agreed to participate, 2 were excluded for missing data and five declined. The sample was comprised of 52 females (36.4%) and 91 males (63.6%). Participants’ mean age was 39.3 ± 11.8. Individuals most frequently reported they were seeking treatment for opioid use (104, 72.7%), alcohol use (68, 47.6%), or cocaine use (55, 38.5%). The majority were stably housed (100, 69.9%) and reported their highest educational attainment to be high school diploma or less (106, 74.2%). Full characteristics are available elsewhere (Hall et al., 2021).

Sample means and ranges for total GBMMS and GBMMS subscales were determined. Mean, minimum and maximum total GBMMS scores were 31.4 ± 9.4, 12.0 and 58.0. The lack of support from healthcare providers subscale (GBMMS LOS) had a mean of 8.2 ± 2.6 and a range of 3–15. The Suspicion subscale (GBMMS SUSP) had a mean of 14.0 ± 5.4 and ranged 6–28. Finally, the Group-based health disparities subscale (GBMMS DISP) mean was 11.8 ± and a range of 4–20.

### 3.2 Correlational analyses

#### 3.2.1 GBMMS total

Spearman's rho analyses yielded moderate, positive, and statistically significant correlations between total GBMMS and original items “I would have gotten addiction treatment sooner if I did not have to worry about racial discrimination by healthcare workers” (hereafter delayed care seeking) (r_s_ (141) = 0.599, *p* < .001), “I would not be surprised if I am discriminated against because of race or color during my addiction treatment” (hereafter anticipation of racial discrimination during addiction treatment) (r_s_ (141) = 0.685, *p* < .001) and “If I am discriminated against during addiction treatment, I will be more likely to relapse” (hereafter concern for discrimination-precipitated relapse) (r_s_ (141) = 0.413, *p* < .001). Weak and positive correlations were found between total GBMMS and “I would not follow the advice of an addiction treatment provider who seemed to hold discriminatory views about my race or color” (hereafter anticipation of discrimination-precipitated disengagement) (r_s_ (141) = 0.353, *p* < .001).

#### 3.2.2 Lack of support from healthcare providers subscale

GBMMS LOS was borderline strongly correlated with anticipation of racial discrimination during addiction treatment (r_s_ (141) = 0.696, *p* < .001). Moderate and positive correlations were found between GBMMS LOS, delayed care seeking (r_s_ (141) = 0.480, *p* < .001), and anticipation of discrimination-precipitated disengagement (r_s_ (141) = 0.425, *p* < .001). Concern for discrimination-precipitated relapse was weakly correlated with GBMMS LOS (r_s_ (141) = 0.391, *p* < .001).

#### 3.2.3 Suspicion subscale

GBMMS SUSP was moderately positively correlated with anticipation of racial discrimination during addiction treatment (r_s_ (141) = 0.677, *p* < .001), delayed care seeking (r_s_ (141) = 0.608, *p* < .001) and concern for discrimination-precipitated relapse (r_s_ (141) = 0.410, *p* < .001). Anticipation of discrimination-precipitated disengagement was weakly correlated with GBMMS SUSP (r_s_ (141) = 0.337, *p* < .001).

#### 3.2.3 Group-based health disparities subscale (GBMMS DISP)

GBMMS DISP was moderately correlated with items anticipation of racial discrimination during addiction treatment (r_s_ (141) = 0.439, *p* < .001) and delayed care seeking (r_s_ (141) = 0.409, *p* < .001). Weak, positive correlations were found between GBMMS DISP, anticipation of discrimination-precipitated disengagement (r_s_ (141) = 0.284, *p* < .001), and concern for discrimination-precipitated relapse (r_s_ (141) = 0.320, *p* < .001).

#### 3.2.4 History of interpersonal racism by healthcare workers

**Fig. 1:**
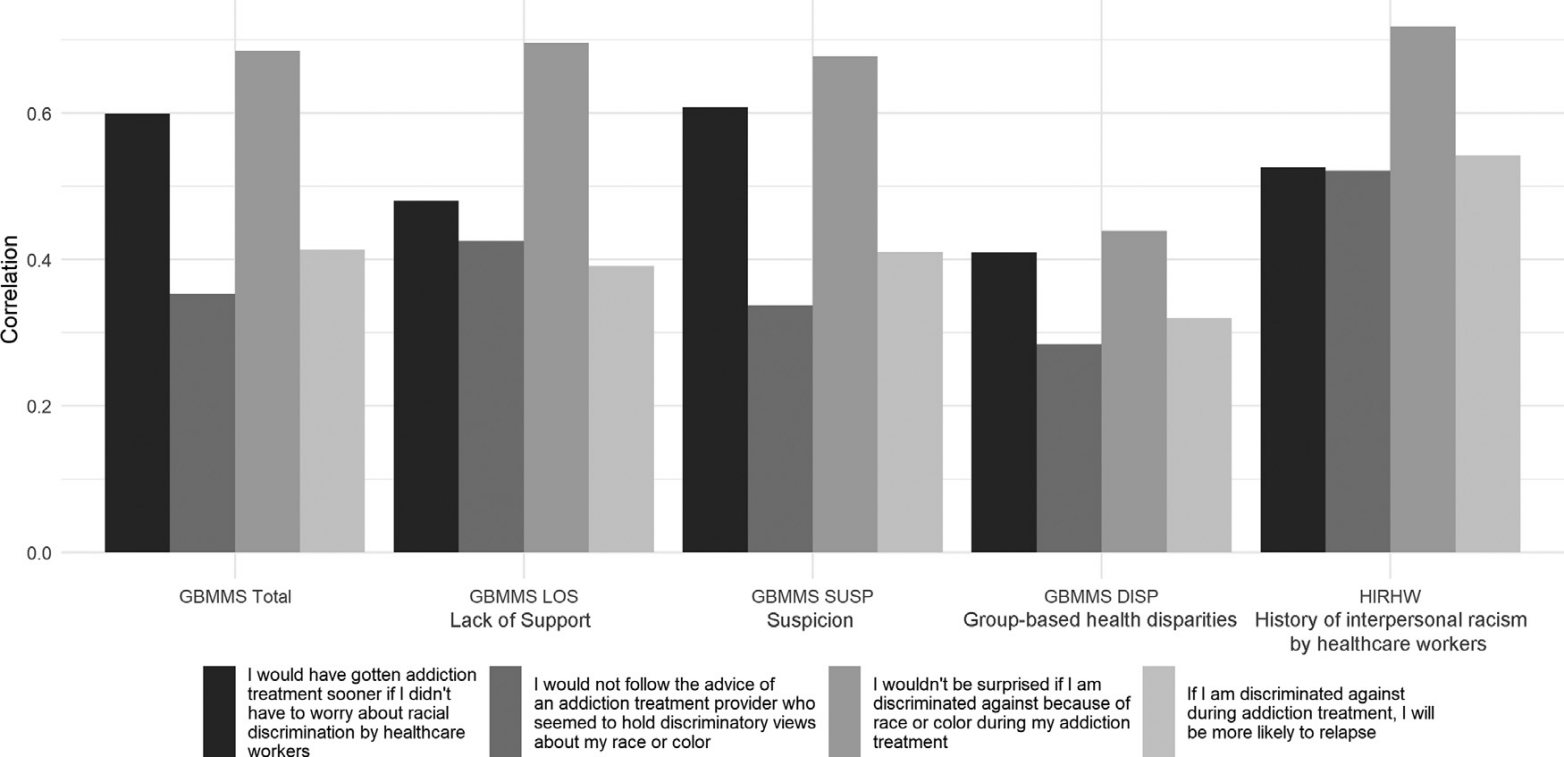
Correlates of group-based medical mistrust (GBMMS), history of interpersonal racism by healthcare workers (HIRHW) and expectations of racial discrimination in addiction treatment.

The statement “I have personally been treated poorly or unfairly by doctors or healthcare workers because of my ethnicity” was strongly correlated with anticipation of racial discrimination during addiction treatment (r_s_ (141) = 0.718, *p* < .001) and moderately correlated with delayed care seeking (r_s_ (141) = 0.526, *p* < .001), anticipation of discrimination-precipitated disengagement (r_s_ (141) = 0.521, *p* < .001) and concern for discrimination-precipitated relapse (r_s_ (141) = 0.542, *p* < .001). Fig. 1 illustrates the correlates of Group-Based Medical Mistrust (GBMMS), history of interpersonal racism by healthcare workers (HIRHW) and expectations of racial discrimination in addiction treatment.

## 4 Discussion

This study provides compelling initial evidence that group-based medical mistrust is associated with self-reported delayed care seeking, anticipation of racial discrimination during addiction treatment, anticipation of discrimination-precipitated disengagement and discrimination-precipitated relapse among Black patients with substance use disorders. All hypothesized associations between medical mistrust and care expectations were confirmed. Total GBMMS, all GBMMS subscales, and the GBMMS item eliciting personal history of medical racism were robustly associated with expectations of racial discrimination and addiction treatment. Delayed care seeking was moderately associated with total GBMMS and subscales measuring perceived lack of support by healthcare workers, suspicion, and awareness of group-based health disparities. Anticipation of racial discrimination during addiction treatment was strongly associated with prior experiences of racial mistreatment during healthcare. Moderate associations with anticipated discrimination were noted with total GBMMS, lack of support by healthcare workers, suspicion and group-based health disparities subscales. Finally, concern for discrimination-precipitated relapse was moderately correlated with total GBMMS, suspicion and personal history of racial discrimination by healthcare workers.

Our findings add to the growing body of literature linking medical mistrust to health inequality across multiple contexts (Benkert et al., 2019). Stereotype threat, or the idea that actions are interpreted by a preexisting framework, usually refers to racially minoritized groups who confirm the negative behavior ascribed to their group (Spencer et al., 2016). Through this lens, past experiences with providers may relate to expectations of discrimination and mistrust; a premise that was largely born out by the data. However, surprisingly, total GBMMS and suspicion and disparity subscales were only weakly associated with anticipation of discrimination-precipitated disengagement. Of all things that were associated with medical mistrust—expecting discrimination, delaying care, concern for relapse—not listening to providers, was the least likely. This finding suggests a possible aperture for engagement whereby there might be a persistent hope and expectation for care, separate from medical mistrust and expectations of racial discrimination, that could provide an opportunity to break the pattern of stereotype threat.

In examining the strongest associations found, a personal history of racism and the suspicion subscale were most strongly correlated with anticipation of racial discrimination during addiction treatment. This finding demonstrates that when entering the therapeutic alliance between patients of color and addiction medicine treatment services it is important to consider personal experiences of racism and associated trauma. Patient-centered communication (partnering with the patient through empathic reflective listening) is an approach that might improve the therapeutic alliance and reduce suspicion (Cuevas et al., 2019). Additionally, although this study did not examine provider race, studies outside of addiction medicine have found patient-doctor race concordance led to greater patient satisfaction scores, presentation to care, and health outcomes(Hill et al., 2018; LaVeist and Nuru-Jeter, 2002; LaVeist et al., 2003). In the United States, the addiction workforce is predominantly White and an increase in addiction treatment providers belonging to under-represented minorities is needed Jordan and Jegede, 2020. Future work should examine the effects of addiction workforce diversity on group-based medical mistrust and addiction treatment outcomes.

Our study was limited by its cross-sectional design which prevented testing correlations between medical mistrust and clinical outcomes. Other limitations center on the unclear generalizability of our findings given the setting (urban Midwestern academic health system serving a large, racially and socioeconomically diverse catchment area) and sample (treatment-seeking participants). It is possible that samples drawn from other geographic locales or from non-treatment seeking populations might differ in medical mistrust and individual and structural barriers to addiction treatment. Future prospective cohort studies should examine baseline GBMMS scores in relation to measurable aspects of addiction treatment such as access, adherence and longitudinal outcomes.

In conclusion, the present study demonstrates that group-based medical mistrust is associated with self-reported delayed care seeking, anticipation of racial discrimination during addiction treatment, anticipation of discrimination-precipitated disengagement and concern for discrimination-precipitated relapse. However, non-adherence to treatment was the least strongly correlated with the GBMMS offering an opportunity for engagement. GBMMS can be utilized by providers to increase understanding of how structural and interpersonal racism stand as important barriers to addiction treatment for Black patients. By addressing these obstacles providers can offer more equitable care.

## Funding

Dr. Hall received funding from the Recognizing and Eliminating Disparities in Addiction through Culturally Informed Healthcare (REACH) program. The REACH Program is made possible by funding to the Academy of Addiction Psychiatry (AAAP) from the Substance Abuse and Mental Health Services Administration (SAMHSA) grant no. 1H79TI08135801. The views expressed in this publication do not necessarily reflect the official policies of the Department of Health and Human Services; nor does mention of trade names, commercial practices, or organizations imply endorsement by the U.S. government. Dr Jordan is supported by grants unrelated to this work (Foundation of Opioid Response Efforts, National Institute on Alcohol Abuse and Alcoholism [R01 AA028778–02], and Yale Center for Clinical Investigation Clinical and Translational Science Award [KL2TR001862]); and from the US National Center for Advancing Translational Science, components of the US National Institutes of Health (NIH), and the NIH Roadmap for Medical Research.

## Role of funding source

Dr. Hall received funding from the Recognizing and Eliminating Disparities in Addiction through Culturally Informed Healthcare (REACH) program. The REACH Program is made possible by funding to the Academy of Addiction Psychiatry (AAAP) from the Substance Abuse and Mental Health Services Administration (SAMHSA) grant no. 1H79TI08135801. The views expressed in this publication do not necessarily reflect the official policies of the Department of Health and Human Services; nor does mention of trade names, commercial practices, or organizations imply endorsement by the U.S. government. The funder had no role in study design; in the collection, analysis and interpretation of data; in the writing of the report; or in the decision to submit the article for publication.

## CRediT authorship contribution statement

**O. Trent Hall:** Conceptualization, Data curation, Formal analysis, Funding acquisition, Investigation, Methodology, Project administration, Writing – original draft, Writing – review & editing. **Nia M. Bhadra-Heintz:** Writing – original draft, Writing – review & editing. **Julie Teater:** Conceptualization, Funding acquisition, Project administration. **Jennifer Samiec:** Visualization, Writing – original draft, Writing – review & editing. **Jose Moreno:** Writing – review & editing. **Kamilah Dixon-Shambley:** Writing – review & editing. **Kara M. Rood:** Writing – review & editing. **David A. Fiellin:** Conceptualization, Methodology, Writing – review & editing. **Ayana Jordan:** Conceptualization, Funding acquisition, Methodology, Project administration.

## Declaration of Competing Interest

The authors declare no conflicts of interest.

## References

[bib0001] Benkert R., Cuevas A., Thompson H.S., Dove-Meadows E., Knuckles D. (2019). Ubiquitous yet unclear: a systematic review of medical mistrust. Behav. Med..

[bib0002] Cuevas A.G., O'Brien K., Saha S. (2019). Can patient-centered communication reduce the effects of medical mistrust on patients’ decision making?. Health Psychol..

[bib0003] Galvan F.H., Bogart L.M., Klein D.J., Wagner G.J., Chen Y.T. (2017). Medical mistrust as a key mediator in the association between perceived discrimination and adherence to antiretroviral therapy among HIV-positive Latino men. J. Behav. Med..

[bib0004] Gryczynski J., Schwartz R.P., Salkever D.S., Mitchell S.G., Jaffe J.H. (2011). Patterns in admission delays to outpatient methadone treatment in the United States. J. Subst. Abuse Treat..

[bib0005] Guerrero E.G., Marsh J.C., Duan L., Oh C., Perron B., Lee B. (2013). Disparities in completion of substance abuse treatment between and within racial and ethnic groups. Health Serv. Res..

[bib0006] Hadland S.E., Wharam J.F., Schuster M.A., Zhang F., Samet J.H., Larochelle M.R. (2017). Trends in receipt of buprenorphine and naltrexone for opioid use disorder among adolescents and young adults. JAMA Pediatr..

[bib0007] Hall O.T., Jordan A., Teater J., Dixon-Shambley K., McKiever M.E., Baek M., Garcia S., Rood K.M., Fielin D.A. (2021). Experiences of racial discrimination in the medical setting and associations with medical mistrust and expectations of care among black patients seeking addiction treatment. J. Subst. Abuse Treat..

[bib0008] Hill, A., Jones, D., Woodworth, L., 2018. Physician-Patient Race-Match Reduces Patient Mortality. SSRN. 10.1016/j.jhealeco.2023.102821 37871470

[bib26] Jordan Ayana, Jegede Oluwole (2020). Building outreach and diversity in the field of addictions. The American Journal on Addictions.

[bib0009] Lagisetty P.A., Ross R., Bohnert A., Clay M., Maust D.T. (2019). Buprenorphine treatment divide by race/ethnicity and payment. JAMA Psychiatry.

[bib0010] Lappan S.N., Brown A.W., Hendricks P.S. (2020). Dropout rates of in-person psychosocial substance use disorder treatments: a systematic review and meta-analysis. Addiction.

[bib0011] LaVeist T.A., Nuru-Jeter A. (2002). Is doctor-patient race concordance associated with greater satisfaction with care?. J. Health Soc. Behav..

[bib0012] LaVeist T.A., Nuru-Jeter A., Jones K.E. (2003). The association of doctor-patient race concordance with health services utilization. J. Public Health Policy.

[bib0014] Manhapra A., Stefanovics E., Rosenheck R. (2020). Initiating opioid agonist treatment for opioid use disorder nationally in the Veterans health administration: who gets what?. Subst. Abus..

[bib0015] Martin C.E., Scialli A., Terplan M. (2020). Unmet substance use disorder treatment need among reproductive age women. Drug Alcohol Depend..

[bib0016] Montgomery L., Burlew A.K., Haeny A.M., Jones C.A. (2020). A systematic scoping review of research on black participants in the national drug abuse treatment clinical trials network. Psychol. Addict. Behav..

[bib0017] Moore A.D., Hamilton J.B., Knafl G.J., Godley P., Carpenter W.R., Bensen J.T., Mohler J.L., Mishel M. (2013). The influence of mistrust, racism, religious participation, and access to care on patient satisfaction for African American men: the North Carolina-Louisiana prostate cancer project. J. Natl. Med. Assoc..

[bib0018] Nina M., Tammy P.H., Tam W., Laura A.S. (2014). Disparities in the use and quality of alcohol treatment services and some proposed solutions to narrow the gap. Psychiatr. Serv..

[bib0019] Samples H., Williams A.R., Olfson M., Crystal S. (2018). Risk factors for discontinuation of buprenorphine treatment for opioid use disorders in a multi-state sample of Medicaid enrollees. J. Subst. Abuse Treat..

[bib0020] Schiff D.M., Nielsen T., Hoeppner B.B., Terplan M., Hansen H., Bernson D., Diop H., Bharel M., Krans E.E., Selk S., Kelly J.F., Wilens T.E., Taveras E.M. (2020). Assessment of racial and ethnic disparities in the use of medication to treat opioid use disorder among pregnant women in Massachusetts. JAMA Netw. Open.

[bib0021] Shelton R.C., Winkel G., Davis S.N., Roberts N., Valdimarsdottir H., Hall S.J., Thompson H.S. (2010). Validation of the group-based medical mistrust scale among urban black men. J. Gen. Intern. Med..

[bib0022] Spencer S.J., Logel C., Davies P.G. (2016). Stereotype threat. Annu. Rev. Psychol..

[bib0023] Street R.L., Makoul G., Arora N.K., Epstein R.M. (2009). How does communication heal? Pathways linking clinician-patient communication to health outcomes. Patient Educ. Couns..

[bib0024] Williamson L.D., Bigman C.A. (2018). A systematic review of medical mistrust measures. Patient Educ. Couns..

[bib0025] Williamson L.D., Smith M.A., Bigman C.A. (2019). Does discrimination breed mistrust? Examining the role of mediated and non-mediated discrimination experiences in medical mistrust. J. Health Commun..

